# Study of Efficacy of a Novel Formative Assessment Tool: Keywords Recall

**DOI:** 10.7759/cureus.69881

**Published:** 2024-09-21

**Authors:** K Venkatesh, D Muthukumar, E Kamala, M Muhil

**Affiliations:** 1 Department of Physiology, Trichy SRM Medical College Hospital and Research Centre, Tiruchirappalli, IND; 2 Department of Physiology, Dhanalakshmi Srinivasan Institute of Medical Sciences, Perambalur, IND; 3 Department of Forensic Medicine, Government Medical College, Tiruppur, Tiruppur, IND; 4 Department of Anatomy, Trichy SRM Medical College Hospital and Research Centre, Tiruchirappalli, IND

**Keywords:** academic performance, novel assessment tool, competency based medical education, summative assessment, formative assessment

## Abstract

Background

Formative assessment is a crucial component of a Competency-Based Medical Education curriculum. Keywords are concise representations of the central ideas and themes explored within a subject. Taking a memory test evaluates knowledge as well as improves future memory.

Aim and objectives

This study intended to study the efficacy of the “keywords teaching” technique and “keywords recall” after a teaching-learning session as an effective tool for formative assessment and the correlation between the performance of students in summative assessments.

Materials and methods

Students of first-year professional faculty of medicine students 2022-23 batch attending pre-clinical (physiology) classroom lectures aged between 18-21 years belonging to both genders who consented to voluntary participation in the study were included in the study. Scores of formative sessions by multiple choice questions, keywords recall assessment tool, and summative sessions were analyzed using paired t-tests. Scores of formative assessments and summative assessments were correlated using Pearson correlation analysis.

Results

Analysis showed formative assessments had a significant (*P *< 0.05) relationship with summative assessment performance. The study indicates a positive correlation between scores for both formative and summative assessments, highlighting the importance of formative assessment in improved academic performance.

Conclusion

Optimal learning can be achieved by testing that emphasizes recall retrieval practice and that is repeated at intervals over time. This study suggests that “keywords recall” after a teaching-learning session is an effective tool for formative assessment.

## Introduction

Medical education primarily focuses on strengthening learner's cognitive frameworks through classroom-based teaching. The field of medical education has seen significant changes over time, transitioning from a conventional, time-oriented structure to a more modern approach based on demonstrated competencies [1]. This transition places greater emphasis on the acquisition of precise skills and information that are essential for medical practice, rather than solely focusing on the completion of a predetermined duration of training. Competency-based medical education (CBME) is acknowledged as a successful method for training medical students [2].

Assessments are an integral part of any curriculum, as they are the key to connecting teaching and learning. Formative assessment is a crucial component of the CBME curriculum since it allows educators to constantly provide feedback, track student progress, and tailor training to match individual learning needs [3]. The ongoing evaluation process includes many methods such as clinical skills assessments, objective structured clinical examinations, and multisource feedback. Formative assessments enable instructors to monitor students' advancement, provide assistance as necessary, and personalize education to meet specific learning requirements. Formative assessment provides valuable feedback to students, allowing them to identify areas of strength and weakness [4].

Factual knowledge is the foundation of how specialists communicate, interpret, and organize their academic discipline, while students must grasp it to understand and solve its difficulties. Information that is considered valuable can be extracted as factual knowledge. Factual knowledge has two subtypes: terminology and precise information and elements.

Words, numerals, signs, and pictures are used in terminology. Labels and symbols are a field's language, allowing experts to communicate. These phrases are used by experts to explain problems and phenomena. Inexperienced learners must grasp the common meanings of labels and symbols. Professionals in the sciences use specialist vocabulary, thus trainees are expected to know more than necessary.

Understanding specific facts, events, locations, people, dates, and sources requires specific details. For their work and analysis, experts rely on this accurate or approximate data. Facts are objective information about books, documents, and sources, while terminology is the language and conventions of a subject. Educators must assess the material's importance and comprehension level. They may prefer students to understand a phenomenon's magnitude or proportion rather than its precise quantification. Educators struggle to assess how well courses incorporate specific information [5].

Keywords, or key phrases, are a limited number of words or phrases that accurately define the overall idea of a work. These keywords also include significant research concepts and approaches [6]. Keywords are concise representations of the central ideas and themes explored within a subject. Taking a memory test evaluates knowledge as well as improves future memory. The process of retrieving material from memory through testing yields learning benefits that surpass those of studying alone, a phenomenon known as the testing effect. Despite compelling and consistent evidence demonstrating that testing enhances the ability to retain and recall material, as well as improves the organization of information in memory, testing remains underutilized as a teaching method by educators and as a self-regulation technique by learners [7].

Medical students need formative assessments to track their development and improve. In formative assessment, medical students might use memory-based learning approaches like concept mapping, where students graphically organize and connect information to better understand and remember crucial concepts; spaced repetition, which improves long-term memory by reviewing information at increasing intervals; self-testing, when students actively retrieve material from memory to assess their knowledge and discover weaknesses; mind mapping, which involves constructing visual diagrams to relate ideas and concepts to improve understanding and retention; usage of acronyms or graphics to remember complex medical concepts or processes.

Medical education is dynamic and rigorous, requiring students to absorb and apply huge amounts of material in real-life situations. Formative assessment shapes medical students' learning experiences. These methods improve students' memory and comprehension of complicated medical subjects. Memory-based learning strategies in formative assessment may help medical students learn more thoroughly and retain it as they experiment with new teaching methods.

Formative assessment that incorporates memory-based learning approaches helps medical students retain the material and understand it better [8]. Students can connect crucial concepts, increase long-term memory retention, actively recall material from memory, visually organize related ideas, and use mnemonic tactics by using these techniques. These methods promote learning and give students the skills they need to apply medical information in real life. This method matches the dynamic and difficult character of medical education, giving students a deeper understanding of key medical knowledge. As educators prioritize new teaching approaches, memory-based learning tactics in formative evaluations may help medical students succeed. Medical students may not benefit from summary, visualization, or keyword mnemonics. To optimize these tactics' effectiveness, alternative ways can be considered. Combining mnemonic techniques with other learning strategies can improve their effectiveness in medical education. Treating mnemonics as retrieval aids rather than core learning strategies can also improve their effectiveness. Testing, active recall, and spaced repetition are key medical student learning tactics in this paper.

However, medical students are sometimes overwhelmed by the amount of factual knowledge they must acquire and don't know how to learn and remember. Paradoxically, testing as an active learning method is more effective than memorizing facts repeatedly. Long-term retention is far higher with frequent testing than with learning. Active recall, or replicating material without cues, is much more effective than passive restudying [9]. Quizzing and trying to remember facts, even if unsuccessful, improve learning. Factual knowledge is better retained when repeated at increasing intervals. Proponents of alternate learning approaches also argue that memorizing may hinder the development of higher-order thinking, clinical reasoning, and problem-solving skills needed for medical practice. Mnemonic devices may cause cognitive overload, especially in the context of fast-increasing medical knowledge and the requirement for adaptive and flexible thinking in clinical decision-making. Some researchers argue that memory-based learning strategies may not work for all medical students due to variances in cognitive styles, learning preferences, and neurodiversity [10]. The obstacles medical students confront in learning facts include too much information. Medical students must acquire a lot of facts about different specialties, which can be daunting and time-consuming. Due to time constraints, pupils struggle to acquire and retain all the facts. Students typically lose facts, making long-term retention difficult.

Optimal learning is achieved by testing that emphasizes recall rather than recognition of material, is repeated at intervals over time, and is accompanied by feedback. This study intended to study the efficacy of “keywords recall” after a teaching-learning session as an effective tool for formative assessment.

## Materials and methods

Study design

The study utilized a cross-sectional study design to investigate the association between formative assessments conducted through traditional class tests by multiple choice questions, formative assessments conducted through a novel tool -"keywords recall" and summative assessments conducted during the academic year between March 2022 and February 2023. ​

Type of study

This was an analytical cross-sectional study.

Study participants

The study included a total of 150 participants consisting of students of first-year professional faculty of medicine students attending pre-clinical (physiology) classroom lectures.​

Inclusion criteria

Students of first-year professional faculty of medicine students attending pre-clinical (physiology) classroom lectures aged between 18-30 years belonging to both genders who consented to voluntary participation in the study.

Exclusion criteria

Students who are not willing to participate are excluded from the study. Students who absent themselves from Physiology lecture classes or summative assessment sessions with attendance less than 75% are excluded from the study.

Ethical clearance

The research study was approved by the Institutional Review Board at Trichy SRM Medical College Hospital and Research Centre, Reference number: 211/TSRMMCH&RC/ME-1/2022-IEC No. : 77 dated 14-03-2022. To secure voluntary participation, participants were provided with a comprehensive explanation of the study's objectives and methods. Before the study, written consent was obtained from all participants, ensuring the confidentiality of information, maintaining respectful conduct, allowing participants to freely enter and exit, and safeguarding the integrity of the data.

Sample size calculation

The sample size was calculated assuming the correlation between e-learning performance and in-course assessment performance values as 0.27 as per the study by Gupta et al. [11]. The other parameters considered for sample size calculation included were power (β) and alpha error (α). The sample size was calculated using the following formula.

$n\geq\left(\frac{Z_{1-\alpha/2\ }+\ Z_{1-\beta}}{\frac{1}{2}\log_e{{\frac{1+r}{1-r}}}}\right)^2+3$

α: 1.96 β: 0.842

The required sample size calculated as per the above-mentioned data was 105. To account for a non-participation rate of about 5% another five subjects will be added to the sample size. Hence the final required sample size would be 110. Since the formative assessment tool introduced is novel, we recruited 150 Bachelor of Medicine and Bachelor of Surgery (MBBS) student participants as they consented to the study voluntarily.

Data collection

As participants, the current study involved first-year medical students of the Tamilnadu Dr MGR Medical University studying at Trichy SRM Medical College Hospital and Research Centre. The class comprised 150 students, with 81 female and 69 male participants. The mean age of student participants was 18.57±0.912 for males and 18.29±0.641 for females (minimum 18 - maximum 21) years. No ethical vote was needed because the study included no medical tests, patient questionnaires, or epidemiological studies. Nevertheless, the study followed Helsinki criteria. The Medical Education Unit of the institution was briefed about the study informally and gave its clearance to conduct it during physiology lecture hours. All of the study participants were told before the project began that they could choose not to participate and that their choice would not affect their future exams or their medical study in general. Participating students signed an informed consent declaration for anonymized participation and publication.

Data collection involved obtaining written responses for formative assessment about the core subject topic taken ​during Physiology lecture hours. There were about 15 formative sessions and eight summative assessment session scores obtained during the academic year excluding the first-year MBBS University exam as it was not conducted during the study period. Of the 15 formative sessions, eight sessions were evaluated through "keywords recall" tool, and seven sessions were evaluated through multiple-choice questions (MCQ). All eight summative assessment sessions were evaluated through long essays, short notes, and multiple-choice questions. The “Key words recall” assessment tool was introduced at the end of the lecture session to record the responses. The responses were evaluated while comparing them with “keywords” answer key which was prepared by the subject expert before the lecture class manually. Matching responses were given a score out of 10. Similarly, the scores obtained by the students in the summative assessments were also followed up for the academic year.

Statistical data analysis

The data were entered in a Microsoft Excel (Redmond, WA, USA) datasheet. Statistical analysis was performed to compare the mean values of scores obtained by students in the formative assessment through the “keywords recall” tool, formative assessment through Multiple choice questions, and regular academic summative assessment sessions. ​ The study intended to evaluate the academic performance of students and ascertain whether there were any notable discrepancies between the formative and summative scores. ​

The continuous variables, including attendance and scores, were represented as the mean value plus or minus the standard deviation. An independent sample 't' test was used to assess the differences in means between the two groups. A Pearson correlation analysis was performed to assess the associations between these factors. For all statistical tests, a p-value of less than 0.05 was considered statistically significant.

## Results

**Figure 1:**
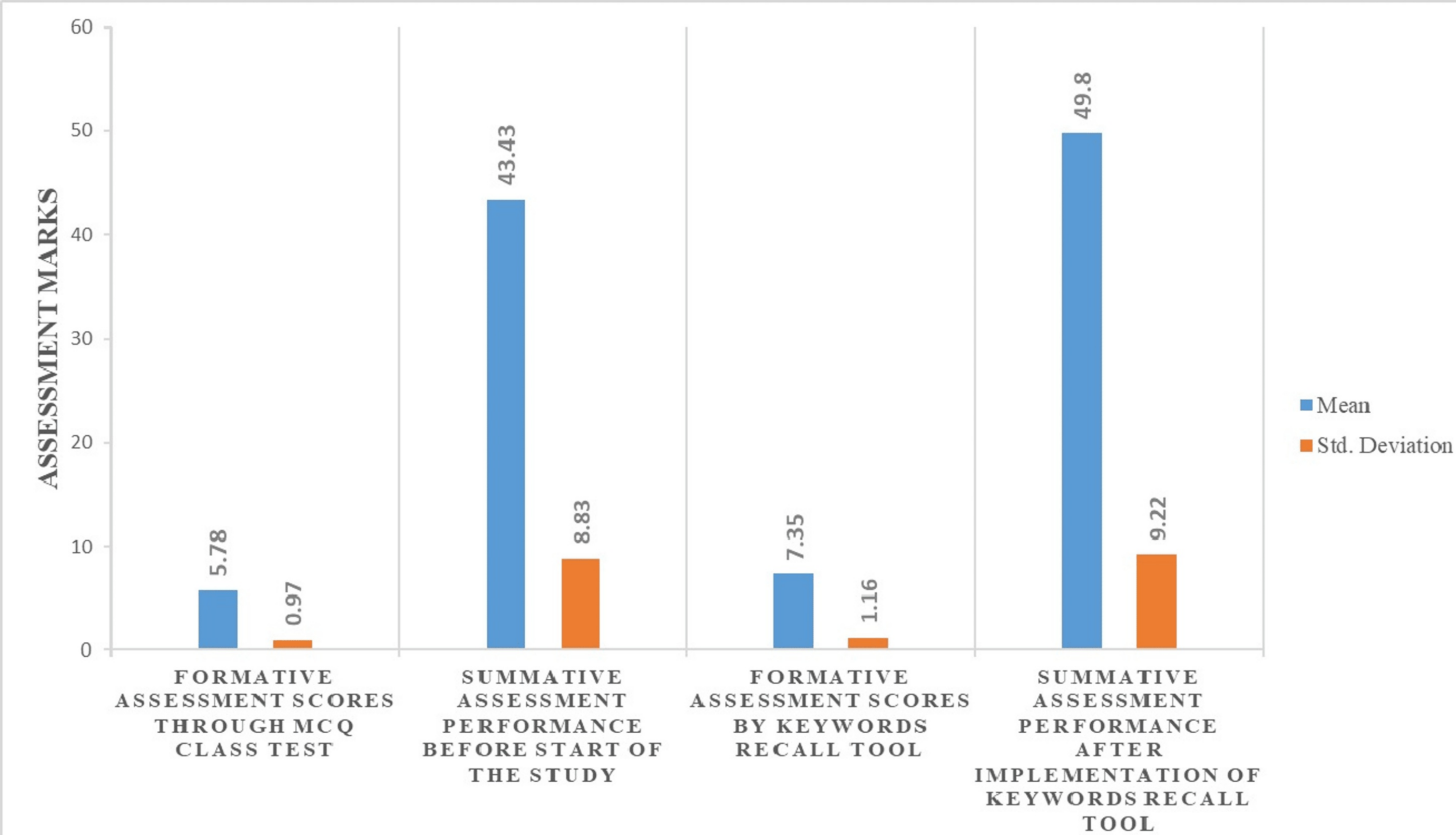
Summary statistics of academic performance during the study

Figure 1 shows the academic performance of participants in the study during the academic year between March 2022 and February 2023. The mean values of formative assessment scores by keywords recall tool (7.35 ± 1.16) were higher than that of multiple choice questionnaire assessment (5.78 ± 0.97) while the mean values of summative assessment that followed up keywords recall formative assessment (49.8 ± 9.22) were higher than that were obtained prior to its implementation (43.43 ± 8.83).

Table 1 showed a statistically significant relationship (r=0.28, p <0.001) between formative assessment scores by multiple choice questionnaire assessment and the summative assessment performance of the participants. Pearson correlation coefficient (r=0.28) indicates low positive correlation.

**Table 1:** Relationship between formative assessment by multiple choice questionnaire (MCQ) test and summative assessment performance * - Statistically significant low positive correlation by Pearson correlation analysis

_Parameter_	_Mean_	_Standard Deviation_	_Standard Error Mean_	_95% Confidence Interval of differences_		_Correlation Coefficient (r)_	_p-value_
				_Lower_	_Upper_		
_Formative assessment score by MCQ test_	_5.78_	_ 0.97_	_ 0.08_	_ 5.62_	_ 5.92_	_ 0.28*_	_ <0.001_
_Summative assessment performance_	_43.43_	_ 8.83_	_ 0.72_	_ 42_	_ 44.86_		

Table 2 shows a statistically significant relationship (r=0.35, p <0.001) between formative assessment scores by Keywords recall and the summative assessment performance of the participants after the implementation of the keywords assessment tool. Pearson correlation coefficient (r=0.35) indicates moderate positive correlation.

**Table 2:** Relationship between formative assessment by keywords recall tool and summative assessment performance * - Statistically significant moderate positive correlation by Pearson correlation analysis

_Parameter_	_Mean_	_Standard Deviation_	_Standard Error Mean_	_95% Confidence Interval of differences_		_ Correlation coefficient_	_ p-value_
				_ Lower_	_ Upper_		
_Formative assessment score by Keywords recall assessment tool_	_ 7.35_	_ 1.16_	_ 0.1_	_ 7.16_	_ 7.54_	_ 0.35*_	_ <0.001_
_Summative assessment performance_	_ 49.8_	_ 9.22_	_ 0.75_	_ 48.31_	_ 51.29_		

## Discussion

The formative assessment sessions focused on a wide range of physiological topics, with varying levels of participant attendance and performance scores. Various competencies related to physiological systems were assessed, such as describing transport mechanisms across cell membranes, red blood cell formation, immunity types, pathophysiology of Myasthenia gravis, muscle contraction, structure of digestive system, conducting system of heart, cardiovascular regulatory mechanisms, mechanics of respiration, cystometry, function tests of the thyroid gland, male reproductive system, and functions/properties of receptors of the central nervous system. The assessments covered knowledge domains. The assessments were at the knowledge level. All sessions were core topics.

There were about seven formative sessions conducted through multiple choice questions slip tests, eight formative sessions through keywords recall assessment tool and eight summative assessment sessions focusing on various physiological systems conducted during the academic year. In our study (Table 1), the mean value of formative assessment scores was 5.78±0.97 and summative assessment scores were 43.43±8.83 before the start of the study. There was a statistically significant relationship between formative and summative assessment scores demonstrated by paired sample t-test (p-value <0.001). The mean value of formative assessment scores assessed using keywords recall assessment tool was 7.35±1.16 and summative assessment scores were 49.8±9.22. There was a significant relationship between formative assessment scores conducted through the keywords recall assessment tool and summative assessment scores by paired t-test (p-value <0.001).

Pearson’s correlation analysis between formative assessment conducted through multiple choice questions and summative assessment scores revealed a low positive statistically significant relationship (r=0.28, p<0.001). While, correlation analysis between formative assessment sessions conducted through the keywords recall assessment tool and summative assessment revealed a moderate positive statistically significant relationship (r=0.35, p<0.001). This finding indicated that keywords recall tool as the end of teaching learning session for formative assessment is more effective in improving academic performance than compared to that of multiple choice questions slip test.

Formative assessment has always been an important part of evaluating and improving academic performance in CBME curriculum. Formative assessment is a continuous assessment measure that is more effective when accompanied by feedback that is given at the earliest. Our study had similar findings of better formative assessment scores resulting in better summative assessment performance conducted by Zhu et al. [12]. Similar to a pilot study conducted by Meher et al. [13], our study obtained a positive correlation between formative assessment and summative assessment scores. 

Kang, McDermott, and Roediger examined how multiple-choice and short-answer questions affected undergraduate students' ability to remember short articles after three days. Short-answer and multiple-choice questions were used on the final exam. The researchers found that students who responded to both short-answer and multiple-choice questions after reading the article had greater information recall on the final test [14]. Our study found that formative assessment with multiple-choice questions improved summative assessment performance.

Karpicke and Blunt conducted a comparison between the effects of retrieval practice and the elaborative study technique of concept mapping in their study. The aim was to evaluate the influence of retrieval practice on the learning of undergraduate-level science topics among students. After examining the effect on individual students, it was found that the students did better on the final exams when they used retrieval practice as a study strategy, compared to using idea mapping [15].

Lyle and Crawford (2011) investigated the impact of retrieval practice on student learning in a statistics course at the undergraduate level. As part of the course, students were directed to allocate the last five to 10 minutes of each class to answer two to four questions that necessitated recalling information about the lecture of the day. The students in this particular segment of the course achieved an average score that was higher on exams throughout the semester compared to students in sections that did not utilize the retrieval-practice strategy. This difference is statistically significant [16]. Similarly in our study, there was a positive significant relationship between formative assessment conducted through keywords recall assessment tool and summative assessment performance.

The limitation of our study may be attributed to the voluntary nature of participant involvement, limited sample size, training of the subject experts regarding the novel assessment tool and the exclusive focus on a specific institution. The study used both formative and summative assessments. However, the "Keywords recall" tool was novel, and the participants had little experience with formative assessments, so the results mostly focused on summative evaluations. The people who took part in this study were first-year MBBS students. It would be helpful to look into how assessment affects the educational growth of postgraduate students in order to make this tool more credible and useful. This study tool is a memory-based retrieval practice assessment tool. Overreliance on memory-based learning may lead to superficial understanding and poor critical thinking in medical students, according to several academics and educators.

## Conclusions

The study found a statistically significant positive correlation relationship between formative assessment and summative assessment performances. Our study also found that keywords recall assessment practice using formative tests had improved learning and performance compared to traditional slip tests conducted through multiple choice questions. Actively taking practice tests raised exam scores, proving that this learning technique works. However, exam pressure makes it harder for students to learn and remember facts. These problems can differ for every student, depending on learning style, study habits, and personal circumstances.

Memory-based learning methods can help students retain and comprehend vital medical information, but they also have drawbacks. Formative assessment should incorporate memory-based learning strategies with careful consideration of the potential downsides and a holistic approach to medical education. By realizing these limits and investigating alternate approaches, educators can maximize memory-based learning techniques while meeting medical students' different needs for comprehensive medical knowledge and skills. In conclusion, medical students can use memory-based learning approaches for formative assessment, but their effectiveness may vary.
